# What is it about coral reefs? Translation of ecosystem goods and services relevant to people and their well-being

**DOI:** 10.1002/ecs2.3639

**Published:** 2021-08-08

**Authors:** Deborah L. Santavy, Christina L. Horstmann, Leah M. Sharpe, Susan H. Yee, Paul Ringold

**Affiliations:** 1 Center for Environmental Measurement and Modeling (CEMM), Gulf Ecosystem Measurement and Modeling Division (GEMMD), Office of Research and Development, U.S. Environmental Protection Agency, Gulf Breeze, Florida 32561 USA; 2 Oak Ridge Institute for Science Education Participant, CEMM, GEMMD, U.S. Environmental Protection Agency, Gulf Breeze, Florida 32561 USA; 3 Pacific Ecological Systems Division, Center for Public Health and Environmental Assessment, Office of Research and Development, U.S. Environmental Protection Agency, Corvallis, Oregon 97333 USA

**Keywords:** beneficiaries, biophysical metrics, coral reefs, final ecosystem goods and services (FEGS), human benefits, human well-being, translation EGS metrics

## Abstract

There is an astonishing diversity of ways in which people benefit from coral reefs. They provide recreation, resource extraction, inspirational, and educational opportunities, among many others as well as being valued just for their existence. As the condition of coral reef ecosystems decline, so do their ability to provide these benefits. Prudent management of coral reefs and the benefits they provide are important as some predict most coral reefs globally will be lost by the mid-21st century. Meanwhile, coral reef managers have limited tools and relevant data to design and implement effective environmental management practices that will enable coral reefs to provide benefits demanded by society. We demonstrate an approach to identify and measure environmental components of coral reefs that directly benefit human well-being. The approach views ecosystems through the lens of a specific set of beneficiaries and the biophysical features directly relevant to each. We call these biophysical features Final Ecosystem Goods and Services (FEGS). In our demonstration, we (1) identify a range of beneficiaries of coral reefs; (2) identify metrics of FEGS for those beneficiaries; and (3) describe how data quantifying those biophysical metrics might be used to facilitate greater economic and social understanding.

## Introduction

Human well-being is reliant upon ecosystems goods and services (EGS) that sustain our society, human health, and economy, and they are often assumed to be available for free (MEA 2005, NRC 2005). The challenge is to ensure environmental issues are considered alongside social and economic consequences when making decisions and policies with competing interests (van Oudenhoven et al. 2018, Hein et al. 2019). Decision and policy makers struggle with how best to protect and manage natural habitats and resources while balancing conflicting interests among a diverse group of human users. Wiser decisions can be made when scientific evaluation of resource condition is linked to the goods and services embraced by the full set of human users and managed by considering their diverse social and economic interests (Thomas et al. 2012, Arkema et al. 2015).

Increasingly, greater emphasis is placed on integrating the full set of benefits when considering decisions that can impact EGS with a growing awareness about the complexity and diversity of connections between natural and human systems. Ecosystems provide goods or tangible biophysical components of nature that provide services to humans (MEA 2005, U.S. EPA 2020). These EGS are critical for decision-making in many contexts; however, the linkages between natural and human systems are complex and multifaceted (U.S. EPA 2015). Many environmental problems are ultimately social problems that require resolving human needs within the limits of ecosystem productivity and resilience (DeWitt et al. 2020). Our approach, which explicitly links ecosystem features to a broad range of human needs greatly facilitates linking ecosystem analysis to social analysis (Tashie and Ringold 2019).

Ecological products and processes directly experienced by human beneficiaries are final ecosystem goods and services (FEGS) (Boyd and Banzhaf 2007, Boyd and Krupnick 2009, Boyd et al. 2016, Tashie and Ringold 2019). Beneficiaries, sometimes grouped into beneficiary classes, are the diverse ways that people use, appreciate, or enjoy nature (Landers and Nahlik 2013, U.S. EPA 2015, Newcomer-Johnson et al. 2020). Final ecosystem goods and services metrics explicitly and directly connect biophysical indicators to the people who directly benefit from them; therefore, a FEGS approach can help reduce ambiguity by providing a framework with clear, direct, and intuitive measurements (Boyd and Banzhaf 2007, Boyd and Krupnick 2009, Boyd et al. 2016, Tashie and Ringold 2019). Because FEGS are the link between biophysical condition and socioeconomic benefits to people, this approach is compatible with other existing socio-ecological systems and frameworks (Elliot and O’Higgins 2020, Piet et al. 2020).

Although the Millennium Ecosystem Assessment (MEA 2005) was pivotal in recognizing the science and attempts to classify EGS, it was not designed to define metrics and identify data needs for quantifying those goods and services in the ways in which they are directly used by people. By focusing directly on this subset of all ecosystem features, FEGS metrics can convey ecosystem status for multiple beneficiaries with common interests and directly link this information for input into further economic and social analyses that are of greatest relevance to people who care about or depend on those ecosystems (Boyd et al. 2016, DeWitt et al. 2020). Additionally, FEGS are an effective communication tool for stakeholders and policy makers to show how people obtain specific benefits from specific biophysical attributes of an ecosystem to prioritize which FEGS are of greatest concern within a given decision context.

The FEGS framework is distinguished from other ecosystem service definitions (MEA 2005, Haines-Young and Potschin 2008) by taking a beneficiary-focused perspective that aims to make ecosystem service analysis more operational by focusing on the causal linkages between biophysical changes and direct measures of social welfare (Boyd and Krupnick 2013). The framework helps to identify FEGS, and it delineates nature into separate ecosystems with boundaries directly linked to specific FEGS and those same beneficiaries (U.S. EPA 2020). Specification of FEGS metrics is important because these are the specific tangible biophysical features or qualities that are needed for management, communication, and social analyses (U.S. EPA 2015). Biophysical scientists use many metrics to understand, describe, and assess ecosystems, but many are not meaningful to laypeople without significant technical translation. In contrast, FEGS metrics represent ecosystems in units that beneficiaries, stakeholders, and decision makers can more easily understand. When FEGS metrics are used in analyses, improvements in the connection between biophysical, social, and economic processes can provide a more accurate assessment of policy changes. When FEGS are represented for a full set of beneficiaries, the analysis can be holistic.

**Fig. 1.**
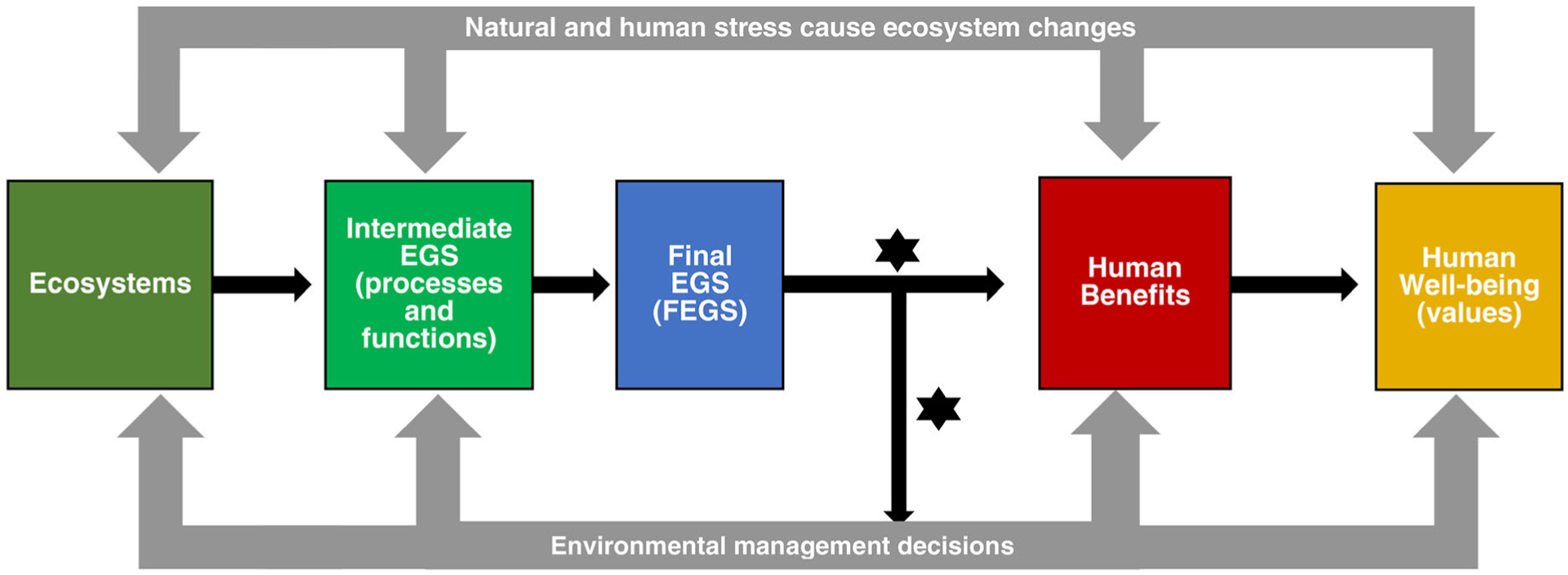
The association between an ecosystem and human well-being relates how ecosystem goods and services (EGS) for use by society are provided by the ecosystem. The ecosystem processes and functions are the intermediary EGS that are used to produce the final ecosystem goods and services (FEGS). Each beneficiary directly interacts with ecosystem attributes that contribute to their human benefits and well-being. FEGS metrics (shown as a bolded star in figure) define qualitative and quantitative terms that describe the linkage to the human benefits and provide a tool to aid in making environmental management decisions (Adapted from Landers and Nahlik 1996, Bruins et al. 2017).

Final ecosystem goods and services serve as the linking metrics to clarify the benefits experienced by people in the specific ways in which they directly interact with ecosystems. Final ecosystem goods and services are contrasted with the broader set of essential intermediate ecosystem goods and services (IEGS) that are required to support or regulate FEGS (Boyd et al. 2016; Fig. 1). The FEGS do not include ecological components or processes required to produce it, these are IEGS (Haines-Young and Potschin 2008). To illustrate the differences between IEGS and FEGS, consider the recreational angler as the beneficiary. The fish is the final good for an angler, whereas the lake is one of the IEGS required to produce the fish they catch. The biophysical metrics for FEGS illustrate how data quantifying biophysical traits of the fish (e.g., species, quantity, health) and its habitat might be used to facilitate greater economic and social understanding (Ringold et al. 2013). Additionally, a FEGS for one beneficiary (e.g., water temperature for an aquaculturalist) may be an IEGS for another (e.g., a recreational angler).

Coral reefs were chosen as one of seven ecosystems examined as part of a larger U.S. national effort developing FEGS metrics using a similar structured process and shared expertise on metric development across these ecosystems (U.S. EPA 2020). Coral reefs were sought out because of their extraordinary biological richness as well as the diverse ways in which people benefit from them. Coral reef ecosystems provide many different EGS that benefit people in diverse ways and have been the subject of increasing study (Carturan et al. 2018, Darling et al. 2019, Hilmia et al. 2019, Williams et al. 2019, Woodhead et al. 2019). Coral reef ecosystems provide many important IEGS and FEGS, such as recreational opportunities for snorkeling and diving, kayaking, sail- or motor-boating, and recreational and subsistence fishing in both developed and developing nations (Moberg and Folke 1999, Yee et al. 2014, 2015). Non-residents and residents alike benefit from tourism opportunities, since the commercialization of SCUBA, millions of divers have paid billions of dollars that sustain local, state, and territorial economies often in developing countries and island nations globally (Cesar 2000, CI 2008, Pendleton 2008, van Beukering et al. 2011, Spalding et al. 2017). Coral reefs also provide food products, aquarium fish, jewelry and curios, personal use products, unique pharmaceutical drugs, and a sense of place, tradition, and culture for local and indigenous peoples (Moberg and Folke 1999, MEA 2005, Principe et al. 2012, Yee et al. 2014, 2015, 2017). Coastline protection from ocean storms and floods for coastal property owners is an important FEGS provided by coral reefs, but it is the coral reef IEGS that linked the final service to coastal protection. Coral reef height and morphology are the biophysical attributes that cause wave attenuation (IEGS) and ultimately the FEGS of shoreline protection. Finally, coral reefs are highly cherished for their existence value for their incredible beauty, high biodiversity, prominent architectural structure, and unique species of fish, invertebrates, corals, and algae.

Protection of ecosystem benefits is important for coral reef managers; a priority heightened by the presence of rapidly increasing coastal human populations increased sea temperatures and ocean acidification (Hughes et al. 2018, Hilmia et al. 2019) increased fishing pressure (Edwards et al. 2014), and the addition of deleterious substances into watersheds and coastal waters (Gardner et al. 2003, Pandolfi and Jackson 2006). Unprecedented losses of up to 90% of the world’s coral reefs are predicted by the mid-21st century (Frieler et al. 2013, Hughes et al. 2018). As coral reef ecosystems decline, their ability to provide valuable EGS is also seriously compromised, impacting human well-being and regional economies (Cesar et al. 2003, Burke et al. 2011, Darling et al. 2019). Meanwhile, coral reef managers and other decision makers remain encumbered by limited tools and relevant data to establish the best ecosystem-based management practices that will enable coral reefs to provide goods and services valued by communities, tourists, recreators, and other private and governmental beneficiaries for the present and in the future (Moberg and Folke 1999, Yee et al. 2015, 2017, Carriger et al. 2019).

Previous studies addressing coral reef EGS have primarily focused on identifying ecological characteristics that contribute to the resistance, recovery, and conservation of ecosystem services based on key ecological traits, life history strategies, and functional ecology of coral reefs (Carturan et al. 2018, Darling et al. 2019, Williams et al. 2019, Woodhead et al 2019). This work is of the utmost importance in understanding coral reefs, but it is not as useful as it might be for describing their status in a way that matters to people and contributes to their well-being. As a result, the application of these frameworks to management of coral reef EGS has been much narrower in context, limited in perspective to ecologists and managers, and focused on identifying coral reef ecological traits and mechanistically relating them to the environmental condition status. Woodhead et al. (2019) use the MEA classification to emphasize a more holistic approach to EGS research by assuming EGS are co-produced by ecosystems and society, and that defining ecological traits in relation to the needs of beneficiaries can provide a deeper mechanistic understanding of implications from disturbances. Unlike the MEA classification (2005), the FEGS framework provides an approach for explicit consideration of the full suite of beneficiaries, particularly when paired with related FEGS classification systems (Landers and Nahlik 2013, U.S. EPA 2015, Newcomer-Johnson et al. 2020), such that key users or relevant attributes are not overlooked.

Our objective was to demonstrate how to apply the FEGS conceptual framework to link people’s well-being to coral reef ecosystems by adopting a user-centric perspective. Coral reefs were chosen as one of seven ecosystems examined as part of a larger U.S. national effort developing FEGS metrics using a similar structured process and shared expertise on metric development across these ecosystems (U.S. EPA 2020). The methods we describe here were refined in partnership with a larger research team interested in FEGS metric application for rivers, wetlands, lakes, estuaries, forests, and agroecosystems (U.S. EPA 2020). We selected beneficiaries, attributes, and metrics that could be useful to coral reef managers to consider when assessing potential decision outcomes. The process can illustrate different potential outcomes useful to reef managers in communicating coral reef status while working with beneficiaries to make trade-off decisions. We described how FEGS are identified, organized, and measured using classification systems to derive meaningful metrics and indicators. The stepwise process consistently (1) defined practical boundaries for the ecosystem of interest; (2) identified selected beneficiaries from a comprehensive list; (3) identified and analyzed ecosystem attributes directly used, appreciated, or enjoyed by each beneficiary, and (4) formulated working hypotheses for proposed biophysical metrics for each beneficiaries (Ringold et al. 2013, U.S. EPA 2020). We provided context to make decisions to determine the types and numbers of FEGS metrics required that are based on beneficiary-based management goals. The list of all potential FEGS metrics for any ecosystem can be quite extensive if the interests of all potential beneficiaries are considered. This manuscript provides a general demonstration of how to use the FEGS framework to allow users and managers to replicate the approach, customize it to their own context, and then test it by vetting metrics with their own beneficiaries in an ecosystem.

## Materials and Methods

We used the FEGS framework (Landers and Nahlik 2013, Ringold et al. 2013, DeWitt et al. 2020, U.S. EPA 2020) to incorporate expert knowledge through a structured process to identify metrics of coral reef FEGS that could be used to identify attributes of direct relevance to human well-being. The national FEGS team was comprised of 18 members (members are in *Acknowledgments*) who were biophysical scientists familiar with the principles of biophysical metric development and selection (McKenzie et al. 1992, Jackson et al. 2000, Dale and Beyeler 2001); ecologists with a broad knowledge and specific expertise from seven terrestrial, aquatic, and marine ecosystems; social scientists; and economists familiar with methods of valuation for both human use and non-use existence values. The FEGS team defined and refined their understanding of FEGS, developed a structured process, and proposed a set of metrics to illustrate application of the FEGS approach (Ringold et al. 2009, 2011, 2013, Landers and Nahlik 2013).

The coral reef metrics team herein are referred to as the metrics team, were a subset of the national FEGS team and were composed of coral reef ecologists (D. Santavy, C. Horstmann, C. Wahle, NOAA) and a social scientist with a specialty in decision science and EGS (L. Sharpe). The metrics team worked to select the beneficiaries, attributes, and FEGS biophysical metrics that related to elements of human well-being (Fig. 1). Metrics were iteratively discussed and reviewed by the larger group of experts on the FEGS team, followed by the metric team refining the metrics as recommended.

Key to the FEGS approach is designating the beneficiaries, then identifying relevant biophysical attributes and how to measure them (Ringold et al. 2013). The following four steps were used to identify metrics of FEGS:

*Step 1:* Delineate ecosystem boundaries;
*Step 2:* Specify beneficiaries and begin to define the final good or service for each beneficiary by asking “What directly matters to that beneficiary?”;
*Step 3:* Select attributes guided by the questions from a standardized list of ecosystem attributes directly used, appreciated, or enjoyed by each beneficiary. Refine ecosystem attributes at the level necessary to support the specification of metrics of the FEGS for each beneficiary; and
*Step 4:* Specify metrics for each beneficiary to develop the FEGS and FEGS metrics using these steps:
  Define the ideal metric;
  Define the available biophysical measures closely related to that ideal metric;
  Use the metrics team expertise to evaluate the ideal metric to determine if the metric(s) proposed sufficiently translate the FEGS into the desired information most easily understood by the beneficiary; and
  Metrics team validate metrics and metrics vetted by FEGS team, review, revise, and repeat until consensus among both groups.

### Step 1: Delineate ecosystem boundaries

Coral reef ecosystems were categorized employing the Final Ecosystem Goods and Services Classification System (FEGS-CS) environment classification (Landers and Nahlik 2013). FEGS-CS is a resource and tool for practitioners that provides a standard classification system for environments and beneficiaries to consistently define, identify, quantify, and value FEGS. (The FEGS-CS has been supplanted by NESCS Plus; Newcomer-Johnson et al. 2020.) A practical definition and clear delineation of coral reef boundaries were determined to clarify what we included and excluded from our consideration. Coral reef and hard bottom boundaries were delineated as hardened substrate of unspecified relief formed by deposition of calcium carbonate from reef-building corals and other stony organisms (relict or live), or existing as exposed bedrock (Kendall et al. 2001). Future practitioners might determine whether benthic habitat maps are available for reefs of interest (e.g., U.S. states and territories use NOAA’s US Coral Reef maps; NOAA CoRIS 2014, NOAA NCCOS 2017) to delimit boundaries and establish a conceptual basis for different uses by beneficiaries of coral reef goods and services.

### Step 2: Specify beneficiaries

The metrics teams attempted to identify all likely beneficiary groups to evaluate the utility of this approach across a diverse spectrum of uses. We included beneficiaries from direct use, indirect use, optional use, and the least tangible non-use value necessary for a total economic benefits analysis (MEA 2005, Turner et al. 2016). We did not select beneficiaries determined to be most important for coral reefs as those can only be identified based on policy or decision context and not by biophysical scientists. Direct use beneficiaries have the most tangible experiences as recreational or consumptive uses. Non-use beneficiaries have the least tangible and most passive experiences as an appreciation for the mere presence of the resource known as existence value. Most non-use beneficiaries never intend to visit or experience the ecosystem, but they highly value the preservation of the resource for future generations also known as bequest value. Because a beneficiary is considered a role or viewpoint rather than a single person or organization, one person might assume multiple beneficiary roles in how they interact with nature (U.S. EPA 2020). For example, an angler might experience the enjoyment of both catching a fish and viewing the beauty of a seascape/landscape provided by the coral reef and shore. In this example, relevant FEGS include catchable fish and enjoying the viewscape. For each beneficiary, we started our process to define FEGS metrics by answering the question “What directly matters to the beneficiary?” to specify what important benefits, uses, or enjoyment were desired by each beneficiary and provided by the coral reef ecosystem. We answered this question with successive levels of refinement to make our thought process transparent.

We chose beneficiaries using the National Ecosystem Services Classification System (NESCS) Plus (U.S. EPA 2015, Newcomer-Johnson et al. 2020) that contained standardized lists of general and specific classes, with descriptions of each general class applicable to any ecosystems (Table 1). NESCS Plus merges two parallel classifications systems FEGS-CS (Landers and Nahlik 2013) and NESCS (U.S. EPA 2015) to leverage their best features to directly link to existing accounting systems for economic valuation and activity (e.g., North American Industry Classification System, NAICS: https://www.census.gov/naics/ last accessed May 2021; DeWitt et al. 2020). Our final beneficiaries were often more detailed than the class of specific beneficiary types defined in the classification system (Ringold et al. 2009, 2013, Nahlik et al. 2012, Landers and Nahlik 2013, U.S. EPA 2015, Newcomer-Johnson et al. 2020) to acquire a finer level of detail required to postulate FEGS metrics. This beneficiary-first approach allows EGS scientists to represent ecosystems in a way that matters to people.

### Step 3: Identify ecosystem attributes

The metrics team identified which ecosystem attributes provide a final good or service for each beneficiary and their defined use by answering “What matters to this beneficiary?” as a heuristic question (U.S. EPA 2020). General features of coral reefs important to each beneficiary were defined as attributes of the ecosystem and first considered at a coarse then a finer level to infer more specificity for appropriate metrics. For example, before entering a reef, a snorkeler contemplates water conditions such as the water quality, clarity, currents, sometimes temperature, and often depth, all attributes desired for a pleasant experience. The FEGS team developed a two-tiered hierarchical classification that is now described in NESCS Plus (U.S. EPA 2015, Newcomer-Johnson et al. 2020) that contains standardized lists of attributes with general descriptions of ecosystem components in Tier 1 and more detailed in Tier 2 (Table 2).

In NESCS Plus, Tier 1 attributes for FEGS are basic components of all ecosystems classified as Water, Air, Weather, Soil and Substrate, Natural Materials, Flora, Fungi, Fauna, and Extreme Events (e.g., fire, flooding, hurricanes), Composite (i.e., multiple single attributes working together, such as landscape aesthetics), all Tier 1 attributes are mutually exclusive except for the last one. Final ecosystem goods and services Tier 2 attributes divided each Tier 1 attribute into multiple and more specific attributes (Table 2). For example, Tier 1 attribute Water was subdivided into the Tier 2 attribute classes of Water quality, Water quantity, and Water movements. Finally, Tier 2 attributes were considered if they should be divided again into sub-attributes that better reflected how and why the attributes were defined as important to each beneficiary. This third tier of sub-attributes was intended to be tailored for each beneficiaries’ interests or use to be best-suited for the beneficiaries unique context, but sub-attributes are not defined in any classification system (e.g., FEGS-CS, Landers and Nahlik 2013; NESCS, U.S. EPA 2015; NESCS Plus, Newcomer-Johnson et al. 2020). Sub-attributes are selected as the final attribute step used by practitioners to translate into FEGS metrics (U.S. EPA 2020).

Each attribute was considered by the metrics team and appraised how well and to what degree the FEGS attribute was appreciated by the beneficiary. We defined how each beneficiary directly interacted with the coral reef by considering all the ways that the beneficiary (Table 1) used, appreciated, or enjoyed attributes using the standardized hierarchical lists classified into Tier 1 and 2 attributes (Table 2). Additional refinement of each Tier 2 attribute into multiple sub-attributes was aided by posing questions as to “What sub-attribute directly matters to each coral reef beneficiary identified in Step 2?” For example, for the aquaculturist beneficiary, we posed the question “Is the water quality sufficient to grow juvenile corals?” and subsequently answered by identifying the Tier 1 attribute as Water, the Tier 2 attribute as Water Quality, and one sub-attribute as Presence of Chemicals and Contaminants. A similar process was followed for each beneficiary and attribute permutation defined by the metrics team. Following selection of the biophysical attributes, the metrics team used sub-attributes to formulate finer scale questions and conceptualized a working hypothesis for each metric. Each question/hypothesis related how natural systems supplied each FEGS to humans, and how the human user directly received (or demanded) the FEGS from nature (U.S. EPA 2020). Deliberations of the following questions guided the process: “What was the desired information wanted by the beneficiary?” and “What metric relayed information that did not need to be translated for the beneficiary?”

### Step 4: Develop FEGS metrics

First, the metrics defined the desired information that lead to a biophysical measurement for each sub-attribute of the ecosystem to identify an ideal metric that was most meaningful to that beneficiary. The metrics team identified metrics that reflected the sub-attributes to embody biophysical aspects of nature that ecologists could measure and monitor directly, often those used for environmental assessment programs. Many potential metrics were scrutinized to select subsets of metrics that were most meaningful to the beneficiaries’ interests. The metrics team considered whether the biophysical metric chosen represented the most apparent, tangible, and intuitive features that resonated with the specific beneficiary. The metrics team described this information so that it would be easily understood by each beneficiary group and ensured the metric was not too technical. Best professional judgment and review of the ecological literature guided the development and identification of the best biophysical measures of reef condition contributing to FEGS (Moberg and Folke 1999, Principe et al. 2012, Yee et al. 2014, 2015, 2020, Albert et al. 2015, Spalding et al. 2017, Beck et al 2018, Carturan et al. 2018, Woodhead et al. 2019).

Frequently more than one metric was suggested for most beneficiaries as they might directly and simultaneously experience or perceive multiple metrics of an ecosystem at the same time. For example, coral reef viewers enjoy the seascape that encompass the sub-attributes of reef type, color, shape, rarity, diversity, richness, and abundance that directly contribute to their appreciation, enjoyment, and usage of the coral reef. The technical metrics and units for each one of these sub-attributes would not have much meaning to a lay beneficiary, rather an indicator of overall pleasure for viewing a coral reef seascape might integrate all or a combination of those sub-attribute metrics to develop a categorical metric or indicator. A rating of excellent, good, fair, or poor seascape viewing experience of coral reefs for beneficiaries might be more meaningful and easier to communicate to a nontechnical user (U.S. EPA 2020). Final ecosystem goods and services metrics can be continuous which are often best for social science or economic analysis or categorial which might be simpler to represent what a beneficiary directly experiences. Often, when biophysical data were not available for the ideal metric, we researched alternative or surrogate metrics from available data sources (Step 4b). While surrogate measures might be the best data available, decision makers must recognize that surrogates might not meet their management goals. This knowledge might help prioritize the collection of data that are a more reliable representation of the FEGS. We evaluated each hypothesis to determine whether the ideal or alternate metric sufficiently translated the final good or service into the benefit that was most easily understood (Step 4c), desired, or most meaningful to that beneficiary.

The proposed FEGS metrics were evaluated by assessing face validity, common sense, and qualitative research (Weber and Stewart 2008, Weber and Ringold 2012, 2019, Weber et al. 2017). Drafts of beneficiaries, attributes, and FEGS metrics prepared by the metrics team were reviewed by the broader transdisciplinary FEGS team comprised of the other 18 ecologists, social scientists, and economists. The FEGS team and metrics team joined to iteratively review and revise until a consensus among both teams accepted the final FEGS metrics as (1) consistent with the FEGS approach being used nationally in other ecosystems, (2) reasonable representation of what was likely to be important to the corresponding beneficiary, (3) clearly defining what was directly perceived by beneficiaries, and (4) measurable (e.g., with temporal and spatial dimensions relevant to decision makers) by ecological and social scientists (Schultz et al. 2012).

The results for a single beneficiary were incorporated into a table designed to guide selection of appropriate metrics for additional FEGS-based assessments that could be conducted for specific decision contexts or locations beyond those identified as exemplified in the U.S. Environmental Protection Agency (U.S. EPA) national effort for FEGS development for seven different ecosystems (U.S. EPA 2020). The table template followed Steps 2–4 as described above, and each table presented a set of examples for FEGS metrics for each of the representative coral reef beneficiaries, selected by the metrics team.

## Results

A diverse spectrum of ecosystems services provided by coral reefs was identified and translated using the FEGS structured framework into related metrics to better facilitate economic and social evaluations for environmental management and policy decisions. The FEGS analysis results for 10 coral reef beneficiaries are in separate tables (Table template Table 3; SCUBA divers and snorkelers Table 4; Anglers Table 5; remaining eight beneficiaries in Appendix S1: Tables S1–S8).

### Step 1: Ecosystem boundaries for coral reefs

Coral reef ecosystems were categorized in the FEGS-CS environmental class: aquatic and a single type of the subclass: Near Coastal Marine. The outer edges of the coral reef architecture generally were delineated by the physical boundaries of the reefs that cannot shift quickly due to the sessile nature and solid calcite structure of reef-building corals. These physical boundaries of the hardbottom reef were appropriate for the SCUBA diver and snorkeler beneficiary. However, decisions about coral reef ecosystem boundaries, delineation, and interpretation became more difficult when considering mobile species, especially for fish desired by angler beneficiaries. The coral reef boundary is more fluid for fish than for sessile reef-building organism and fish freely swim to adjacent ecosystems such as mangroves, seagrass beds, and open ocean. However, the physical boundaries of coral reefs were still considered representative of where experienced boat captains could anchor to increase the likelihood that anglers would catch desired fish species. Another exception encountered was for coastal property owners who did not directly benefit from the FEGS on or above the reef, but instead tens of meters to kilometers away the shoreline where the property was located. Yet, the biophysical attributes relevant to coastal protection, such as wave attenuation over the reef, were within the physical bounds of our study (Sheppard et al. 2005, Ferrario et al. 2014). Furthermore, most beneficiaries require a boat to access the physical boundaries of the reef in order to experience or extract FEGS. As a result, physical factors in the vertical water column over the physical boundary of the reef that influence the experience of being in a small to medium-sized boat over the reef were also considered within the boundary delineation, such as safety issues related to access, surf, tides, and weather conditions.

### Step 2: Beneficiaries of coral reefs

Using the NESCS Plus (Newcomer-Johnson et al. 2020), the metric team identified 10 classes of beneficiaries that experienced potential benefits from coral reef ecosystems: Agriculture; Commercial/Industrial; Government, Municipal, and Residential; Commercial/Military Transportation; Subsistence; Recreational; Inspirational; Learning; Non-use; and Humanity (Table 1). The tenth class, Humanity, is considered inclusive of all humans, and thus members of all other beneficiary classes (not analyzed here). We identified 17 beneficiary subclasses, excluding the general categories of Other Recreational and Other Inspirational that enjoy, consume, or use coral reef FEGS out of a total of 38 subclasses in NESCS Plus (Table 1, coral reef subclasses bolded). The 10 beneficiary groups analyzed were SCUBA divers and Snorkelers; Anglers; Coastal property owners; Learners; Inspirational users; Non-users; Boaters and Kayakers; Ornamental Extractors; Pharmaceutical Extractors and Bioprospectors; and Aquaculturists.

The metric team demonstrated the flexibility of the FEGS framework by grouping several angler subclasses with significant overlap in their interests and attributes by consolidating them into one beneficiary group. Beneficiary classes and subclasses consolidated were Subsistence class, Food subsisters subclass; and Recreational class with two subclasses Catch and release or Catch and keep. We restricted Anglers to those who used hook and line or small nets to fish. There are additional subclasses that can be analyzed such as spear fishermen or commercial extraction, or alternative groupings following the same process to accommodate and develop more detailed metrics for specific applications or locations. Our combined angler group serves to illustrate how multiple beneficiary classes with overlapping interests may be combined to make operationalization more efficient; however, we also illustrate how as separate subclasses their interests may differ.

Learners, Inspirational user, and Non-use classes were only considered at the class level. Learners valued the health of the reef and studied specific aspects including assessment, measurements, and monitoring activities, a role performed by educators, students, and researchers. Inspirational users cared about the overall health of the coral reef from artistic (artists, photographers, videographers included by metrics team), cultural, spiritual, and ceremonial perspectives. Finally, Non-use beneficiaries cared about the existence of coral reef ecosystems in the present and future. Other beneficiaries analyzed were Ornamental Extractors using live reef organisms for display in aquariums or dead for jewelry or decorative products; Commercial or Industrial users focusing on Pharmaceutical Extractors using organisms for medical, cosmetic, and beauty products; and Aquaculturists rearing juvenile or adult corals for multiple purposes such as aquaria trade and reef restoration.

### Step 3: Ecosystem attributes for coral reefs

Tier 1 attributes assigned for coral reefs were Water, Soil and Substrate, Flora, Fauna, and Extreme Events, Composite; Tier 2 attributes and sub-attributes developed for coral reef beneficiaries are bolded text in Table 2. In several cases, the sub-attributes selected by the metrics team were not always unique but were relevant across several different beneficiaries. For example, beneficiaries who required surface contact or complete immersion into the sea to experience the FEGS benefit, shared the Tier 2 attribute Water quality while desiring to select safe and healthy locations. These sub-attributes were developed by considering how Water quality influenced the benefit if water contact was dangerous or unhealthy for beneficiaries especially if Chemicals and contaminants, Pathogens and parasites, and Water clarity (sub-attributes of water quality) were present and negatively impacted the health of beneficiaries (Table 2). A Tier 2 attribute experienced by the Learner, Inspirational, Non-use, and Pharmaceutical beneficiaries (Table 2) were Fauna or Flora community. Learners, Inspirational, and Non-use beneficiaries would appreciate general reef health, like high percentages of coral cover and abundance to heighten their seascape experience, whereas the Pharmaceutical beneficiary would prefer a high diversity of fauna and flora for their biochemical interests.

### Step 4: FEGS metrics for coral reefs

For this step, we detail the development FEGS metrics for two beneficiary classes to illustrate the application of the FEGS approach: recreational SCUBA divers and snorkelers, and anglers who catch fish on coral reefs. Results for the other eight beneficiaries are found in Appendix S1: Tables S1–S8.

#### SCUBA divers and snorkelers.—

The metric team limited analysis of SCUBA divers and snorkelers (now referred to as divers) to those who were primarily interested in recreational diving or snorkeling (i.e., not for commerce, research, salvage, spearfishing, or treasure hunting). Their interests posited as questions were “Will my dive be enjoyable and safe?” and “Is the environment appealing?” (Table 4, Step 2) to explicitly illustrate what directly mattered and was most important to them. Tier 1 attributes selected were Water, Fauna, Flora, Soil and Substrate, and Composite (Table 4, Step 3a). For each Tier 1 attribute, multiple Tier 2 attributes included Water quality and Water movement, Charismatic fauna, Faunal community, Floral community, Substrate quality, and Composite environmental aesthetics. Each Tier 2 attribute had multiple sub-attributes which specifically defined the benefits, interests, or uses desired by divers (Table 4, Step 3b). For example, direct linkages between the attribute tiers and sub-attributes (Table 4, Step 3c) identified for Water quality were visibility, and chemicals and contaminants found in the water column, and for Water movement were currents and wave intensity. More detailed information was obtained by asking a finer scale question, such as “Is there sufficient visibility to be pleasurable for divers?” (Table 4, Step 4a). Analysis ended when the ideal and actual biophysical metrics proposed were the same or no data were available for the ideal metric and the next best metric was identified (Table 4, Steps 4b–d).

Fourteen FEGS and metrics were developed for divers (Table 4), after being vetted by the FEGS team, who collectively have engaged in recreational and scientific SCUBA diving for thousands of hours on reefs. Additionally, the FEGS and metrics were presented in at least one but as many as nine published scientific studies. Results from published surveys ranked divers most desired attributes as water clarity, coral community, and fish community when deciding whether and where their dive would be enjoyable and safe (Flores-de la Hoya et al. 2018). The FEGS metrics for water clarity, detailed in Table 4, Step 4b, cited water visibility was the most preferred metric by cited by divers and measured by diver observation or Secchi disk depth (Leeworthy and Wiley 1996, Ramos et al. 2006, Uyarra et al. 2009, Flores-de la Hoya et al. 2018, Leeworthy et al. 2018). Local scale observational data are not regularly reported, and visibility can be variable depending on the location, season, time of day, and ocean conditions. Estimation of Secchi depth transparency from satellite data as Kd values or chlorophyll a makes these data regularly available and over larger spatial extents (Kulshreshtha and Shanmugam 2015).

The presence of chemicals and contaminants in the seawater was identified as a critical sub-attribute to determine “Is the water quality high enough to be safe for diving?” (Table 4). Recreational divers must discern health risks as they are fully immersed in seawater, and the most stringent health risk standards must be communicated to them. The ideal indicator integrates multiple metrics to communicate safe exposure levels that are easily interpreted by users to determine whether it is safe to dive by considering the presence and concentrations of fecal matter, human pathogens, toxins, chemicals, or other harmful contaminants (Table 4). Other potential hazards could be sea conditions including current flow (e.g., “Is the water moving too fast?”) and wave height (e.g., “Are waves dangerous for divers?”) were identified as important FEGS metrics a diver would use to determine if their dive would be enjoyable and safe. Marine advisory reports on wave height may be the most accessible data easily understood by divers (Table 4, Step 4d).

Fish and coral community metrics reflecting what was most desired by divers were overall abundance; presence of rare species; biodiversity; species richness; size; color and unique behaviors and morphologies, most of which are surrogate metrics. A multimetric index expressed as a simple categorical indicator would be easier for divers to decide whether their dive would be enjoyable. Literature identifies the amount of live coral cover as the second most common coral community metric associated with making dives pleasurable (Pendleton 1994, Williams and Polunin 2000, Uyarra et al. 2005, Kirkbride-Smith et al. 2013, Schuhmann et al. 2013, Flores-de la Hoya et al. 2018) with coral colony abundance a close second (Shafer 2000, Wielgus et al. 2010, Paterson et al. 2012, Polak and Shashar 2013). For fish communities, fish abundance was the most preferred metric cited (Leeworthy and Wiley 1996, Shafer 2000, Williams and Polunin 2000, Wielgus et al. 2003, Uyarra et al. 2005, 2009, Polak and Shashar 2013), followed by fish size (Shafer 2000, Williams and Polunin 2000, White 2008, Uyarra et al. 2009, Paterson et al. 2012, Giglio et al. 2015, Flores-de la Hoya et al. 2018). Divers preferred and were attracted to large (e.g., turtles, dolphins, sharks), colorful (e.g., butterfly-fish, wrasses, sponges, sea fans), and/or unusual marine charismatic organisms (e.g., trunk fish, eels, Christmas tree worms). Most studies evaluating how much viewing of charismatic fauna contributed to the divers’ pleasure did not specify what metric they were using unless it was either abundance or presence (Uyarra et al. 2005, Schuhmann et al. 2013). Important sub-attributes for flora community were the presence of charismatic algae (i.e., colorful algae, unusual shapes), or nuisance and harmful algae (i.e., algal blooms or toxic species; Bauman et al. 2010). Substrate quality indicative of reef structure emphasized the divers’ preference for surface complexity such as large spur and groove formations, tall coral structures with complex caves, swim through caverns, and grand underwater viewscapes (Musa et al. 2006). Metrics used for reef structure were reef structural complexity (Williams and Polunin 2000, Kirkbride-Smith et al. 2013) and reef topography (Ramos et al. 2006, Flores-de la Hoya et al. 2018) which were usually measured as rugosity and reef height.

#### Coral reef anglers.—

The angler beneficiary merged three angler types: recreational catch and release and catch and eat, and subsistence anglers. We assumed anglers fished from boats and had minimal contact with the seawater, posing no health concerns from contaminated seawater exposure. Anglers were interested whether “Is this a good place to go fishing?” and “Will the boat be enjoyable and safe to fish from?” (Table 5).

**Fig. 2.**
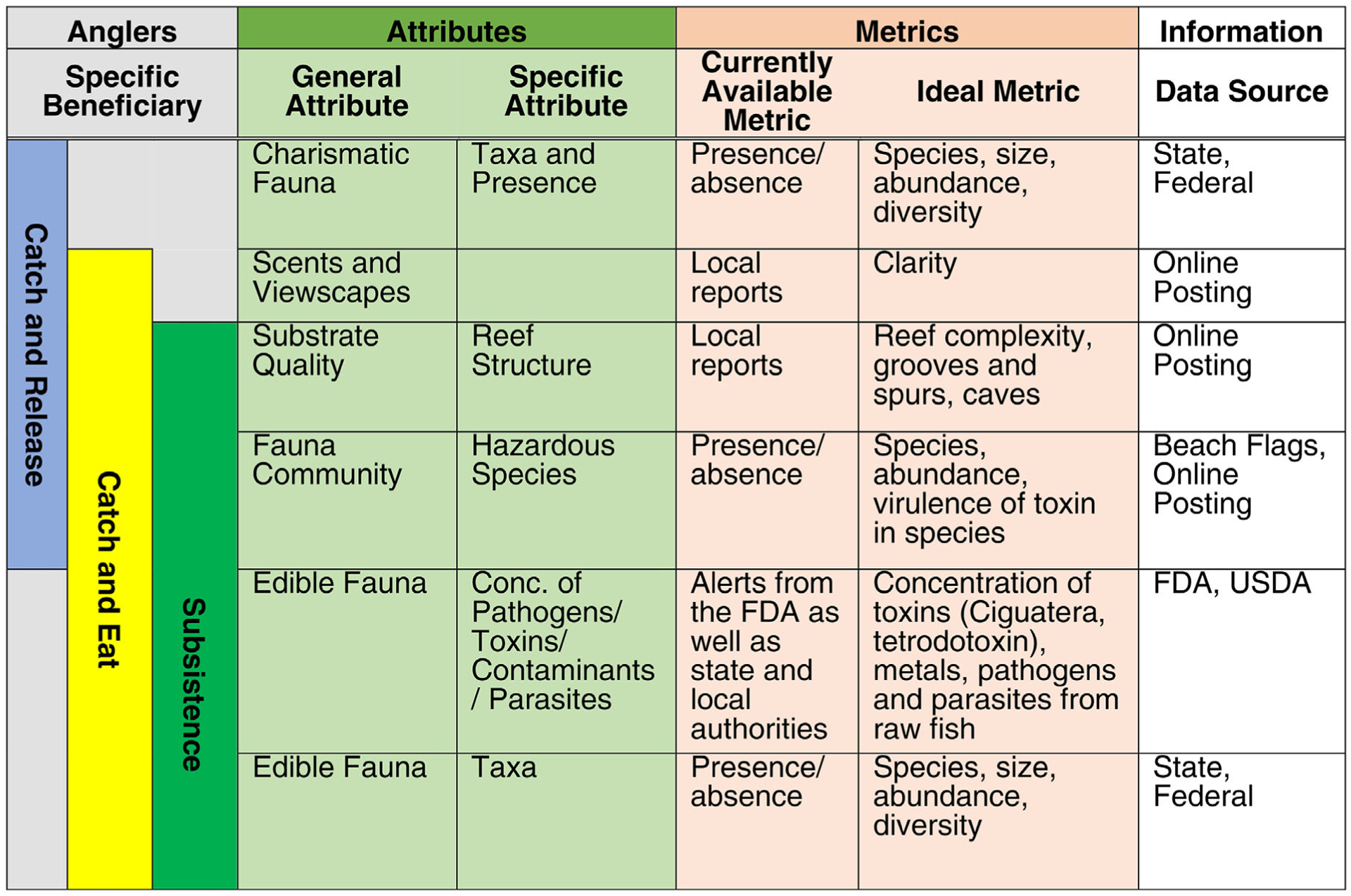
Common overlap of important and consequential final ecosystem goods and services (FEGS) attributes and metrics for all coral reef anglers who extract fish with hook and line or small hand nets for personal enjoyment or subsistence food.

The angler subclasses overlapped in their interest in Fauna (Table 5, Step 3), with distinctions in metrics that reflected different priorities within each subclass. The catch and release angler was primarily interested in charismatic fish species that possessed widespread popular appeal, greater challenges to land (i.e., fighting fish, e.g., tarpon), or symbolic value (i.e., prized species, e.g., marlin; Fig. 2). Ideal metrics could include presence, abundance, size, diversity, and species of available fish (Table 5, Step 4b). The catch and eat angler cared about the same fish attributes as the catch and release angler, but their preferences might be limited to species and lengths based on those suitable for consumption (e.g., grouper, snapper, and tuna) or of legal keep size. Catch and eat and subsistence anglers were more concerned about effects of potential biological or chemical contaminants from consumption (Table 5, Step 3c) than the catch and release angler (Fig. 2). The subsistence angler cared about the fish species and the consumers’ safety, but their highest priority was to catch the most and largest fish that was safe for his family to eat with the least effort. Ideal metrics for fauna communities for each angler type are in Table 5. Other important attributes for anglers included substrate quality and composite environmental aesthetics (Table 5, Step 3). Recreational anglers were assumed to care more about composite environmental aesthetics than subsistence anglers (Fig. 2). Seascape or viewscape, measured in terms of water clarity and other aesthetics, was assumed to be most relevant to the appeal of a site to recreational anglers (Table 5, Step 4).

#### Beneficiaries with cross-cutting FEGS metrics.—

**Fig. 3.**
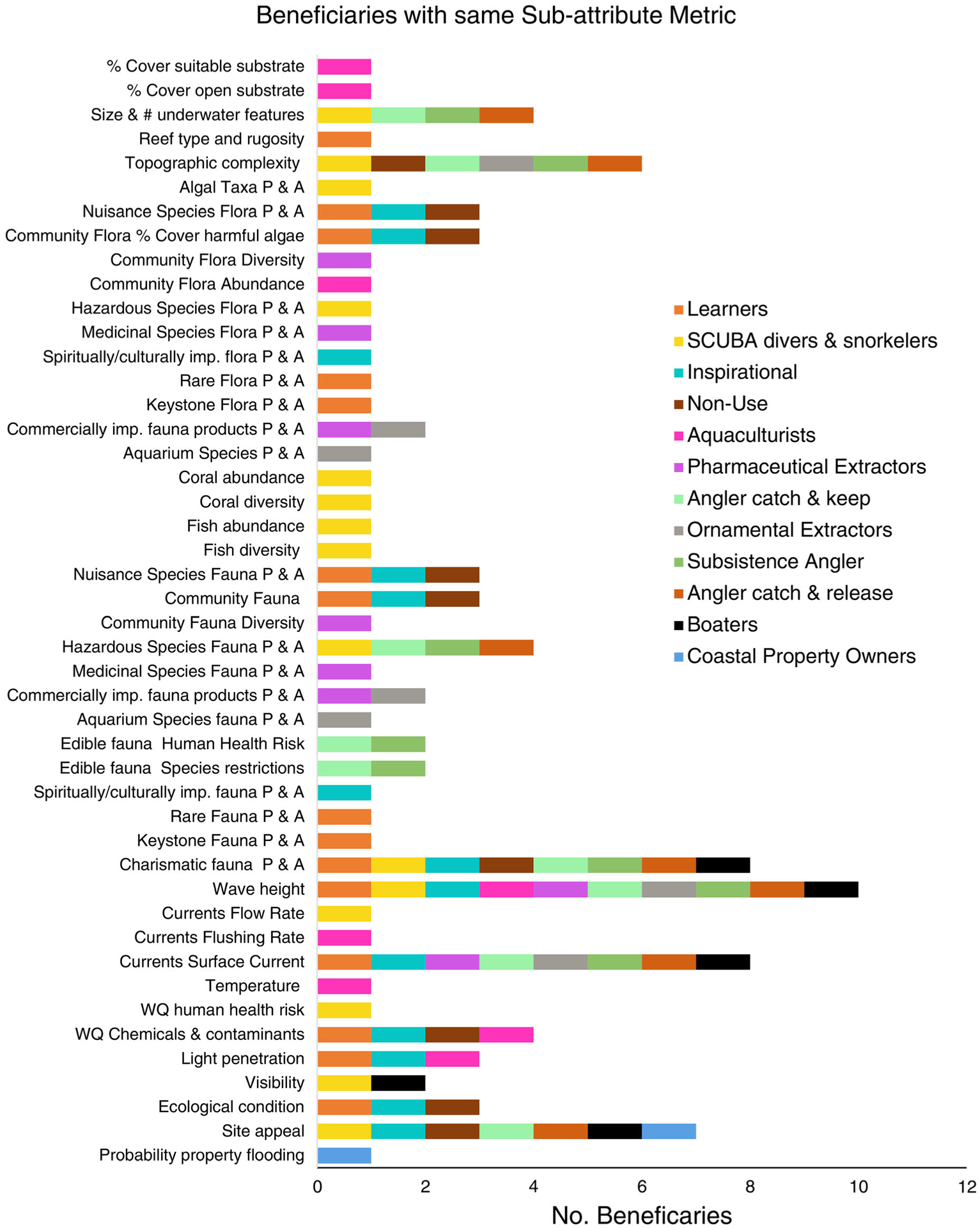
Bar graph showing the final ecosystem goods and services (FEGS) sub-attributes and metrics identified for each beneficiary. Wave intensity and wave height followed by the presence and abundance of charismatic fauna were the highest cross-cutting metric among the beneficiaries we analyzed. P & A, presence and abundance; WQ, water quality.

There were many FEGS metrics which crosscut the spectrum of 12 coral reef beneficiaries as each angler subclass was considered individually for this exercise. Both wave intensity and current strength metrics were identified for 10 of the 12 (83%) beneficiaries for coral reefs (Fig. 3). The most obvious rationale was that almost all beneficiaries must travel to the reef by boat and remain in the boat for the duration of experience, often anchoring on or by the reef dependent on whether they were divers, anglers, or extractors. If users experienced high waves and strong currents, they would likely postpone or cancel their trip or seek another location to enjoy coral reef FEGS. Consequently, there was significant overlap between Tier 2 attributes important to Boaters (Appendix S1: Table S5) and Anglers (Table 5). Sub-attributes associated with an enjoyable and safe boating experience for Water Movement (Tier 2) with their associated metrics in order of preference were wave intensity using indicators of wave height, speed, and direction; and water currents using indicators of tidal phase, weather, wind speed, and wind direction (Table 5). Only the Coastal Property Owners and the Non-use beneficiary with interests in existence and bequest values did not travel to the reef to directly “use, appreciate, or enjoy” the reef. The next set of FEGS identified as important to most beneficiaries were experiential that brought the pleasure and satisfaction of seeing charismatic fauna, viewscapes or lovely underwater gardens, and grandiose reef structures.

## Discussion

The suite of FEGS metrics identified for beneficiaries exemplify the wide diversity of ways stakeholders use and benefit from coral reefs (Appendix S1: Tables S1–S8). We demonstrated the application of the FEGS framework for 10 beneficiary groupings of coral reef ecosystems (U.S. EPA 2020). We did not intend for beneficiaries or FEGS metrics presented here to illustrate final or most appropriate to all coral reef locations or applications. They serve as a starting point and require additional formulation for decision context, vetting by decision makers and beneficiaries, and customization as needed to assist decision makers for determining the best fit for their issues. The identification of beneficiaries, or even the interests of the same beneficiaries, might differ greatly depending on location (Caribbean vs. Indo-Pacific regions), scale (local decision vs. national policy), and priorities of ecosystem services used in developed and developing nations (subsistence vs. recreational uses for extraction of resources). The FEGS framework and process can be broadly adapted and expanded to identify alternative beneficiaries as needed, as well as to tailor attributes and metrics to local issues, users, and stakeholders.

We developed our FEGS metrics using the hierarchical NESCS Plus classification to exemplify selection of the beneficiaries, attributes, FEGS, and biophysical metrics after defining the context using general and specific questions. It is likely that our selected elements may differ considerably, be less familiar, or of less importance for others with different management responsibilities. In those cases, there might not be consistent criteria to propose or select FEGS metrics on a comprehensive basis, so surrogate metrics can be substituted. In other cases, direct measurement of some attributes might be difficult or expensive, justifying the use of surrogate metrics that best approximate valued attributes while acknowledging inherent limitations of using surrogates.

Coral reef managers desire tools and approaches to assist them in problem definition and finding solutions, because many have very limited resources, time, and expertise to make important decisions. Ecosystem-based management aims to guide local and regional experts to organize and streamline the level of information required to formulate the desired results and identify trade-offs and uncertainty in predicting ecosystem outcomes while weighing socioeconomic concerns against ecosystem condition (Sharpe et al. 2020). Final ecosystem goods and services concepts can be integrated at many points along the decision process to incorporate ecological, social, and economic interests that aim to balance conservation goals to maintain functioning ecosystems with different EGS desired by conflicting or differing socioeconomic values and interests of stakeholders (Russell et al. 2020). Use of the FEGS approach can provide managers with plain language to directly link environmental concerns to the community’s values. A values-focused decision process (Gregory et al. 2012) can help guide decision makers to focus discussions on the most relevant information that matters about a decision. Clarifying “what really matters” can prevent collecting the wrong information for the wrong problem which can lead to irrelevant or misleading assessments (Carriger et al. 2019). This increased focus on what stakeholders’ value might increase the likelihood of greater support for final decisions across more of the community because it has considered their priorities (Gregory et al. 2012). The FEGS framework facilitates a values-focused process by helping to identify measurable objectives that are directly relevant and meaningful to stakeholders (Yee et al. 2017).

Those identifying our current time as the Anthropocene Era have proposed that the trajectory of change imparted by humans is irreversible, and scientists must acknowledge that the forces of human impacts and intervention are rapidly changing the structure and function of reefs (Pendleton et al 2016, Williams et al. 2019, Woodhead et al. 2019). Reports of coral reef degradation emphasize the importance of integrating expertise of social scientists and economists to link tangible attributes of natural systems to human well-being and increase the combined legitimacy of their decisions (Thomas et al. 2012, Arkema et al. 2015, Yee et al. 2015). They advocate that the forces of nature alone are no longer controlling our coral reef ecosystems in the Anthropocene. A new paradigm must integrate human well-being, social, cultural, and economic processes with ecological theory in addition to using traditional biological, geological, and physical processes that have always been central to the study of ecosystem relationships at large spatial and temporal scales (Ellis 2015, Österblomet al. 2017, Williams et al. 2019). Ecosystem condition and EGS are increasingly influenced by human socioeconomic and cultural drivers, such as global trade, markets and finance, vast human migration to the coasts, and behavioral choices associated with increasing demands on all resources (Hicks et al. 2016). Much of the current coral reef research has focused on measuring the decline of coral reef ecosystems in response to these socioeconomic and cultural drivers, but little has been done to consider a broader scope of EGS that incorporates them a priori in measurable and interpretable information (Kittinger et al. 2012, Norström et al. 2016). The FEGS framework was created for such circumstances to begin to link human influences and economic principles with ecosystem condition and those services available.

The FEGS framework can be adapted to many different applications, additional beneficiaries, and scaled up or down both spatially and temporally as required by decision needs of the environmental manager or communities. The identification of beneficiaries and FEGS linking metrics can be tailored to the local scale for a specific ecosystem and period of time. Examples include streams (Ringold et al. 2013) or seasonal variability in water clarity of lakes (Angradi et al. 2018). However, a regional or national status and trends report might summarize a broader, more general set of outcomes, benefits, and beneficiaries, making FEGS more useful to apply to areas where impacts might be made. It might be desirable to parse beneficiaries more finely as we have done with the recreational SCUBA divers and snorkelers, and as suggested in Ringold et al. (2013). Depending on the context, the number of beneficiaries and associated metrics can quickly escalate to numbers that are impractical to implement; in fact, our limited demonstration identified dozens of potentially relevant metrics. Managers can focus their efforts on the most meaningful issues that appeal to the widest array of beneficiaries and most sensitive to potential management actions. However, having a complete and holistic view of the relevant metrics will allow managers to select those they choose to focus on more deliberately if there are limited funds for monitoring or assessment focusing on metrics that are meaningful to multiple beneficiaries could be a cost-effective approach. The FEGS Scoping Tool (FST) has been developed to aid managers to prioritize such approaches to be used in conjunction with the FEGS metric development approach presented here. The FST provides a transparent means to prioritize stakeholders, develop beneficiary profiles, and choose among ecosystem attributes as those of shared importance to the community (Sharpe and Jenkins 2018, Sharpe et al. 2020), prior to identification of metrics. Final ecosystem goods and services metrics can be useful for regulatory purposes to evaluate alternate management actions such as risk assessment endpoints (Munns et al. 2015); integrate into other decision support models (e.g., Envision, VELMA; McKane et al. 2020); compare outcomes of alternative management options on ecosystem services; design restoration and revitalization strategies for cleanup of contaminated sites (DeWitt et al. 2020); study resiliency after natural disasters; restore large ecosystems; and even examine different future climate change scenarios on coral reefs.

A primary advantage of the FEGS framework is the ability to be very flexible for operationalizing EGS from a beneficiary perspective, particularly when paired with standardized and hierarchal classification systems (Newcomer-Johnson et al. 2020) that can be adopted to specific decision contexts, spatial scales, or locations. It has the potential to take existing EGS analyses of coral reefs performed from an ecologist’s perspective (Carturan et al. 2018, Darling et al. 2019) and provide the link to show how ecological outcomes influence beneficiaries’ preferences that can be directly connected to evaluate social welfare or economic outcomes (Boyd and Krupnick 2013). Unfortunately, insufficient data availability for marine ecosystem service measures can be a barrier to operationalizing the ecosystem service concept (Culhane et al. 2020). Our analysis shows that there is existing information that can be applied to ecosystem service assessments for coral reef ecosystems for some ideal biophysical measurements most relevant to beneficiaries, but in other cases reasonable proxies may need to be substituted.

The conceptual framework for ecosystem goods and services continues to be expanded and incorporated into decision-making by governmental, national, and international organizations as these entities better define their values. Boyd and Banzhaf (2007) formalized the FEGS concept, and it continues to grow in application. A goal of ecosystems services research is to continue development of the FEGS framework to increase its utility, efficiency, and make it more broadly applicable to social scientists, communities, and environmental managers. As FEGS are subject to continuing refinement, the results will enable increasing collaborations between natural and social scientists to understand FEGS and how humans value them. Improvements in the application of the framework could define more useful and relevant data, leading to better-informed decisions for the management of all ecosystems. We encourage future practitioners to further define and refine the metric(s) to better represent the benefits received by the beneficiary as alternative data sources and metrics emerge. Our work focused on static biophysical metrics that matter to people, but additional research would improve application to scenarios applied over multiple temporal and spatial scales by incorporating measures of FEGS metrics in the design of modeling, monitoring, assessment, and reporting programs.

## Supplementary Material

Supplement1

## Figures and Tables

**Table 1.** FEGS beneficiary categories from NESCS Plus (U.S. EPA 2015, Newcomer-Johnson et al. 2020) hierarchical classification to identify beneficiaries who directly use, interact with, or directly perceive nature.

General beneficiary	Specific beneficiaries	Beneficiary description	Coral reef beneficiary
**Agricultural**	Livestock grazers; agricultural processors, **aquaculturists**; farmers; foresters; other agricultural beneficiaries	Beneficiaries who use the environment for agricultural or forest production activities	**Aquaculturists**
**Commercial/industrial**	**Food extractors; timber, fiber, and ornamental extractors**; industrial processors; private energy generators; **pharmaceutical and food supplement suppliers**; fur/hide trappers and hunters; private drinking water plant operators; **commercial/industrial property owners**	Beneficiaries who directly use the environment for industrial or commercial production activities not included in the other categories	**Ornamental extractors; pharmaceutical extractors**
**Government, municipal, and residential**	Municipal drinking water plant operators; **residential property owners**; public sector property owners; military/coast guard; public energy generators	Governmental, military, and residential beneficiaries who directly use the environment in ways not included in other categories	**Residential property owners**
Transportation	Transporters of goods; transporters of people	Military and commercial beneficiaries who use the environment as media to transport goods or people	
**Subsistence**	Water subsisters; **food and medical subsisters**; timber, fiber, and fur/hide subsisters; **building material subsisters**; other subsisters	Beneficiaries who use the environment to support subsistence activities	**Subsistence angler**
**Recreational**	**Experiencers and viewers**; food pickers and gatherers; hunters; **anglers; waders, swimmers, and divers; boaters; other recreational uses**	Beneficiaries who use the environment to support recreational activities	**SCUBA and snorkelers; boaters; angler catch and release; angler catch and keep**
**Inspirational**	**Spiritual and ceremonial participants and participants of celebration; artists (photograph/videographers),**† **other inspirational**	Beneficiaries use or appreciate the environment as a source of inspiration	**Inspirational users**
**Learning**	**Educators and students; researchers**	Beneficiaries who directly use the environment for educational or scientific research activities	**Learners**
**Non-use**	**People who care (existence); people who care (option/bequest)**	Individuals who benefit from the environment in ways that do not require or are not associated with direct use of or contact with a final ecosystem good	**Non-use**
**Humanity**	**All humans**	Everyone, regardless whether they actively recognize or appreciate the final ecosystem good, because they are available to everyone and used by everyone to live (e.g., air for breathing)	

*Notes:* FEGS, final ecosystem goods and services; NESCS, National Ecosystem and Services Classification System. Beneficiaries chosen by the metrics team for this study of coral reef ecosystems appear in boldface.

† For consideration of coral reef ecosystems, we included photographers and videographers as artists.

**Table 2.** FEGS attribute classification developed as a two-tiered hierarchal architecture derived from NESCS PLUS (U.S. EPA 2015, Newcomer-Johnson et al. 2020, Sharpe et al. 2020).

Tier 1 Attribute (coarse scale)	Tier 2 Attribute (coarse scale)	Sub-attribute (fine scale)
Basic elements of all ecosystems that comprise all aspects of any one ecosystem. Tier 1 attributes are what the beneficiary is interacting with (related to the user role). All are distinct except “composite” and “extreme event” tiers, that are derived from multiple component categories	Tier 2 attributes are related to how the beneficiary is interacting with Tier 1 attribute (related to the use itself). Aspects of each basic component that the beneficiaries are directly concerned with. All aspects of each component should be represented	Specific examples of the Tier 2 attribute for which metrics can be developed
**Water**	**Water quality**	**Chemicals and contaminants** **Pathogens and parasites** **Clarity** **Visibility** Salinity **Temperature**
	Water quantity	Water depth Water level Water flow
	**Water movement**	**Wave intensity** **Wave duration** **Wave magnitude** **Currents**
Air	Air quality	
Weather	Wind strength/speed Precipitation Sunlight Temperature	
**Soil and substrate**	Soil quantity Soil quality **Substrate quantity** **Substrate quality**	
Natural materials	Fuel quality Fuel quantity Fiber material quantity Fiber material quality Mineral/chemical quantity Mineral/chemical quality Presence of other natural materials for artistic use or consumption (e.g., shells, acorns, honey)	
**Flora**	**Flora community**	**Species richness; measure of abundance or community function? Presence of invasive/nuisance species**
	**Edible flora**	**Is it present? Percent cover? Sufficient abundance for ecosystem function, extractive use, condition?**
	**Medicinal flora**	**Same as edible flora**
	**Keystone flora**	**Same as edible flora**
	**Charismatic flora**	**Same as edible flora**
	**Rare flora**	**Same as edible flora**
	**Commercially important flora**	**Same as edible flora**
	**Spiritually/culturally important flora**	**Same as edible flora**
Fungi	Fungal community	Species richness; measure of abundance or community function? Presence of invasive/nuisance species.
	Edible fungi	Is it present? Percent cover? Sufficient abundance for ecosystem function, extractive use, condition?
	Medicinal fungi	Same as edible fungi
	Rare fungi	Same as edible fungi
	Commercially important fungi	Same as edible fungi
	Spiritually/culturally important fungi	Same as edible fungi
**Fauna**	**Fauna community**	**Species richness; measure of abundance or community function? Presence of invasive/nuisance species.**
	**Edible fauna**	**Is it present? Percent cover? Sufficient abundance for ecosystem function, extractive use, condition?**
	**Medicinal fauna**	**Same as edible fauna**
	**Keystone fauna**	**Same as edible fauna**
	**Charismatic fauna**	**Same as edible fauna**
	**Rare fauna**	Same as edible fauna
	Pollinating fauna	Same as edible fauna
	Pest predator/depredator fauna	Same as edible fauna
	**Commercially important fauna**	**Same as edible fauna**
	**Spiritually/culturally important fauna**	**Same as edible fauna**
**Extreme events and composite**		
**Flooding**	**Risk of flooding**	
Fire	Risk of fire	
Extreme weather events	Risk of extreme weather events	
Earthquakes	Risk of earthquakes	
**Environmental aesthetics**	Sounds Scents **Viewscapes** Phenomena (e.g., sunsets, northern lights, etc.)	
**Naturalness**	**Ecological condition**	
Open space	Acreage	

*Notes:* The sub-attribute column was developed by the metrics team to refine Tier 2 for coral reef ecosystems. Each attribute pertaining to the coral reef ecosystem appears in boldface. FEGS, final ecosystem goods and services.

**Table 3.** The table format template used to present all steps of FEGS metrics development.

Beneficiary category	Category name from NESCS Plus		Sub-category	Sub-category not always from NESCS Plus		General beneficiary description	Beneficiary description from NESCS Plus and other pertinent details	
Information comes from	NESCS Plus		Social scientists + ecologists					
Step 2	Step 3a	Step 3b	Step 3c	Step 4a	Step 4b		Step 4c	Step 4d
What matters directly to this beneficiary? (FEGS)	Attribute 1	Attribute 2	Sub-attribute (fine scale)	Desired information (metric hypothesis)	Ideal biophysical metric (underlying desired information)	Available biophysical metric (unit)	Translation of ideal biophysical data to desired information	Metric
An example for an angler “Is there a reasonable chance that I can safely catch a fish in an appealing place?”	Tier 1 attribute from NESCS Plus (Table 2). Basic elements of all ecosystems that comprise all aspects of any ecosystem. Tier 1 attributes are what the beneficiary is interacting with (related to the user role)	Tier 2 attributes from NESCS Plus (Table 2). It relates how beneficiary is interacting with Tier 1 attribute (related to use itself). Aspects of each basic component that the beneficiaries are directly concerned with. All aspects of each component should be represented	Specific aspects of the Tier 2 attributes for which metrics can be developed. These are not standardized but developed at discretion of those defining metrics	Finer scale question, what is it about this attribute that matters to the beneficiary? Hypothesis formulation	What are the ideal biophysical data that underpin the desired information? For example: “What biophysical data are needed to be translated into a metric that are meaningful to beneficiaries?”	If the ideal biophysical data are not available, what data could be used instead. If the ideal data are available, enter the same information as in previous column	Describe how the available biophysical data in previous column are translated to the desired information in column E. Sometimes no translation is required from the ideal vs. available biophysical metric	Identify the best biophysical metric that answers questions in 1st and 5th columns. If ideal data not available use general metric that can be refined by specific beneficiary later

*Notes:* FEGS, final ecosystem goods and services. Information includes the beneficiary category, the sub-category, and a general beneficiary description for all those included in this role. The listed attributes represent those selected by the metrics team that were important for illustration sake, with the acknowledgment that the details will differ depending on the issues, partners, and decision context. Examples are provided for each category. Attributes and beneficiaries were selected from NESCS Plus classification system (National Ecosystem and Services Classification System [NESCS]-Plus; U.S. EPA 2015, 2020, Newcomer-Johnson et al. 2020).

**Table 4.** Formulation of FEGS metrics for the SCUBA diver and snorkeler beneficiary, primarily interested in recreationally SCUBA diving and snorkeling (i.e., not for research, salvage, spearfishing, or treasure hunting) on shallow-water tropical coral reefs.

What matters directly to this beneficiary? (FEGS)	Attribute Tier 1 (coarse scale)	Attribute Tier 2 (coarse scale)	Sub-attribute (fine scale)	Desired information (metric hypothesis)	Ideal biophysical metric (underlying desired information)	Available biophysical metrics (units)	Translation of ideal biophysical metrics to desired information	Biophysical metric
Step 2	Step 3a	Step 3b	Step 3c	Step 4a	Step 4b		Step 4c	Step 4d
Will my dive be enjoyable and safe?	Water	Water quality	Visibility	Is there sufficient visibility to be pleasurable to divers?	Turbidity values, Secchi disk, Satellite imagery, light meter	Turbidity: FTU and NTU, ppm. Visibility: m. Satellite chlorophyll a: relative concentrations. Light penetration: Kd, PAR	Secchi disk measurements translate to visibility, but turbidity and light penetration need to be translated to ft or m	Visibility
			Chemicals and contaminants	Is WQ sufficient to be safe for diving?	Fecal matter, pathogens, and toxins	Coliforms, enterococci, vibrios (CFUs). Microbial toxins, heavy metals. chemicals (μmol/l)	Use EPA recommended standards to translate into human health risks	Human health risk
		Water movement	Currents	Is the water moving too fast for beginner divers or snorkelers?	Currents of the water surrounding the reef	Flow rates	Risk of drifting away or getting pushed against rocks	Flow rate
			Wave intensity	Are waves dangerous for divers in the water	Wave height, speed, and direction	Wave height, speed and direction	Marine advisory report based on wave speed, direction, height	Wave height
	Fauna	Charismatic fauna	Charismatic fauna abundance	Do these species attract the beneficiary?	Presence, abundance	Presence, abundance	Presence of fauna directly increases appeal	Charismatic fauna presence and abundance
		Fauna community	Hazardous Species	Is there a chance hazardous species will pose a risk to beneficiary?	Species, abundance, virulence of toxin in species	Species, abundance	Abundance of toxic species increases potential of contact with diver	Presence and abundance
			Fish diversity	Do these species attract beneficiary?	Species, size, color, richness, rarity, unique behavior, and morphology	Biomass, size, diversity, richness, species name, feeding guilds, species description	Big, colorful fish in high abundance translates directly to user appeal. Unique morphology and behavior increase preference	Fish diversity
			Fish abundance	Does the amount of species attract beneficiary?	Amount, rarity	Abundance	Same as fish diversity	Fish abundance
			Coral diversity	Do these species attract beneficiary?	Species, size, color, richness, rarity, unique behavior, and morphology	Percentage of live coral cover, species name, morphotype, richness, size (cm), health, rugosity	Colorful, large colonies of various species in high abundance translates directly to appeal. Unique morphology increases preference	Coral diversity
			Coral abundance	Does the amount of species attract the beneficiary?	Amount, rarity	Percentage of live coral cover, species name, morphotype, abundance, size (cm), health, rugosity	Same as coral diversity	Coral abundance
	Flora	Flora community	Algal taxa	Are there interesting algae species present?	Rarity, color, size, amount of, unique morphology	Abundance, species name, size, diversity, percent cover	Species with unique morphology and color are appealing	Algal presence

*Notes:* FEGS, final ecosystem goods and services. This beneficiary recreates with total emersion into the sea, so by definition, this beneficiary has contact with water. The second-row references what step number in our procedure the results represent. The listed attributes represent those selected by the metrics team that were important for illustration sake, with the acknowledgment that the details will differ depending on the issues, partners, and decision context.

**Table 5.** Formulation of FEGS metrics for coral reef anglers, primarily interested in angling by hook and line or small net on shallow tropical coral reefs.

What matters directly to this beneficiary? (FEGS)	Attribute Tier 1 (coarse scale)	Attribute Tier 2 (coarse scale)	Sub-attribute (fine scale)	Desired information (metric hypothesis)	Ideal biophysical metric (underlying desired information)	Available biophysical metric	Translation of ideal biophysical data to desired information	Biophysical metric
Step 2	Step 3a	Step 3b	Step 3c	Step 4a	Step 4b		Step 4c	Step 4d
Is this a good place to go fishing?	Water	Water movement	Wave intensity	Is it safe to go out?	Wave height, speed, and direction	Wave height, speed, and direction	Marine advisory report based on wave speed, direction, height	Wave height
			Currents	If in a boat, do I have to anchor?	Tide, weather, wind speed, and direction	Tides, wind speed, and direction	Marine advisory report based on wind speed, direction, tides	Flow rate
	Fauna	Charismatic fauna	Fish taxa	Will I catch what I am expecting?	Species, size, abundance, diversity	Species, size, abundance, diversity	Large, edible species translates to angler expectation for good fishing	Species name, presence
		Edible fauna†	Same as charismatic fauna	Same as charismatic fauna	Same as charismatic fauna	Same as charismatic fauna	Same as charismatic fauna	Same as charismatic fauna
		Edible fauna‡	Pathogens/toxins/contaminants/parasites	Will there be a chance of sickness when eating caught fish?	Toxins (Ciguatera, tetrodotoxin), metals, pathogens, and parasites from raw fish and contaminants	Concentration of contaminants in tissue	Fish health indicator complies contaminant information for human food safety	Human health risk
		Fauna community	Hazardous species	Will hazardous species get caught or scooped in net and pose threat to beneficiary?	Species, abundance, virulence of toxin in species	Species, abundance	Presence of toxic species increases potential of contact with angler	Presence and abundance
	Soil and substrate	Substrate quality	Reef structure	Will hook or net get stuck?	Reef complexity, grooves, and spurs, swimthroughs, caves	Reef type, rugosity	Complex reef structure increases potential for loss of gear	No. and size U/W features and topographic complexity
Is the environment appealing?	Composite	Environmental aesthetics§	Viewscape	Is this reef aesthetically enjoyable?	Color of water, algae, clarity and smell, lack of sound	Field crew opinion, Secchi depth, algal abundance	Opinion of field crew reflects angler’s preference	Site appeal

*Notes:* FEGS, final ecosystem goods and services. This beneficiary is a composite of both recreational and subsistence anglers who catch and release or catch and eat fish, or who catch fish for food to sustain themselves and families. The beneficiary has minimal contact with water. The second-row references what step number in procedure the results represent. The attributes selected by the metrics team were important for illustration sake, with the recognition that details will differ depending on the issues, partners, and decision context.

† Different based on specific beneficiary.

‡ Not high priority for “catch and release” anglers.

§ Not high priority for subsistence anglers.
