# Supplementary material for: What is it about coral reefs? Translation of ecosystem goods and services relevant to people and their well-being
Source: Ecosphere. Author manuscript; Available in PMC 2022 Aug 8. (PMC8686212; doi:10.1002/ecs2.3639)
Supplement: Supplement1 [file NIHMS1753686-supplement-Supplement1.pdf]

Supporting Information
Appendix S1

Ecosphere. What is it about coral reefs? -- Translation of ecosystem goods and services relevant to people and their well-being. DL Santavy, CL Horstmann, LM Sharpe, SH Yee, and P Ringold

The tables in this Appendix provide metrics derived for eight additional beneficiaries that directly interact with the coral reef ecosystem and are not addressed in the publication: residential property owners (Table S1); ornamental extractors (Table S2); pharmaceutical extractors (Table S3); aquaculturists (Table S4); kayakers, paddleboarders and boaters (Table S5); non-users (Table S6); learners (Table S7); and inspirational beneficiary (Table S8). The coral reef ecosystem, beneficiaries, and attributes are classified using the National Ecosystem Services Classification System—Plus (NESCS Plus) (Newcomer-Johnson et al. 2020). Conceptually, this classification system considers how state and Federal agencies that interact with the environment are organized and how these agencies design monitoring programs.

"This work focuses on metrics and principles for national and regional scales of analysis. As such, the metrics and the reports based on them are expected to be more useful for agents acting on behalf of collections of individuals as they interact with ecosystems, not necessarily individuals as they make decisions on a day-to-day basis. Having said this, the process and the results that our ecosystem experts went through, and the metrics identified, should be a useful starting point for those focusing on community scales of analysis." (US EPA 2020)

The beneficiaries, attributes, and final ecosystem goods and services (FEGS) metrics for each coral reef-specific beneficiary are organized in comprehensive tables. These beneficiary-specific tables report the biophysical metrics for the FEGS. The structured process for proposing metrics used the following Steps 2-5 and the table format template (See Table 3 in manuscript).

Step 1. Ecosystem Delineation: explain how biophysical scientists bound ecosystems for practical purposes.
Step 2. Beneficiary Specification: describe the beneficiaries to be considered for each ecosystem. These were adapted from a classification provided by the companion National Ecosystem Services Classification System—NESCS Plus (Newcomer-Johnson et al., 2020) (See Table 1 in manuscript).
Step 3. Attribute Specification: identify the biophysical components of nature (e.g., flora, fauna, water) that links with the ecosystem service and beneficiary's interests. The attributes were drawn from a hierarchical list provided in NESCS Plus (See Table 2 in manuscript).
Step 4. Metric Specification: describer the units of the attribute and discuss the difference between ideal and available metrics.
Step 5. Data Availability: consider the availability of appropriately scaled data for the ideal metric and propose alternative metrics when extensive, spatially explicit data are not available. These alternative metrics often represent surrogates or proxies for the ideal metric; it is important to note the limitations of surrogate data.

We aimed to use the FEGS tool to provide results for additional beneficiaries that directly benefit from coral reef ecosystems. The results can be applied to improve the capacity of biophysical scientists to

work with social scientists to improve coral reef ecosystem management and understand how policy changes might affect them. The applications of the FEGS tool is reliant on the quality of the underlying data. The data were obtained from published literature and EPA reports, websites, or other sources provided. Importantly, most of the data used in this report are used to illustrate concepts rather than to provide quantitative conclusions.

References

Newcomer-Johnson, T., Andrews, F., Corona, J., DeWitt, T., Harwell, M., Rhodes, C., … Van Houtven, G. (2020). *National Ecosystem Services Classification System (NESCS Plus)*. (EPA/600/R-20/267). U.S. Environmental Protection Agency.

Sharpe, L., Hernandez, C., & Jackson, C. (2020). Prioritizing stakeholders, beneficiaries and environmental attributes: A tool for ecosystem-based management. In T. Higgins, M. Lago, & T. DeWitt (Eds.), Ecosystem-based Management, Ecosystem Services and Aquatic Biodiversity: Theory, Tools and Applications (pp. 189–212). Amsterdam: Springer.

US EPA (U.S. Environmental Protection Agency). (2020). *Metrics for national and regional assessment of aquatic, marine, and terrestrial final ecosystem goods and services*. EPA645/R-20-002. U.S. Environmental Protection Agency. https://www.epa.gov/eco-research/national-ecosystem-services-classification-system-nescs-plus.

Table S1: FEGS and metrics for Residential Property Owners.

**Beneficiary Category**	14.0303 Residential Property Owners	**Sub-Category**	Coastal Property Owners
**General Beneficiary Description**	While changes in property value are not a FEGS, residential property owners are affected by the environment in which their property resides. This could be homeowners or businesses on the coast. The basic principle of coastal protection by coral reefs is the observation that reefs dissipate wave energy either by wave breaking or friction by reef structures		

**What Matters Directly to this Beneficiary?**	**Attribute 1 (Coarse Scale)**	**Attribute 2 (Coarse Scale)**	**Sub-Attribute (Fine Scale)**	**Desired Information**	**Ideal Biophysical Data (Underlying Desired Information)**	**Available Data (and Unit)**	**Translation of Ideal Biophysical Data to Desired Information**	**Metric**
Will my property be damaged?	Extreme Events	Risk of flooding	Intensity	Will the speed of the waves damage the property?	Water depth, bathymetry, historical wave height, previous damage. Coastline features soil type, slope, elevation, vegetation (e.g., mangroves)	Data: areas vulnerable to flooding, friction factor, wave model, coral reef typology, wave energy dissipated by reef type, & land use values	Risk of damage to my property including docks & boats from wave forces	Wind Speed
			Duration	Will the duration or length of the waves impact the property?				Wave length
			Magnitude	Will the height & width of the waves impact the property?				Wave height
			Predictability	Can beneficiary predict when flooding will occur?	Tides, storm frequency, coastline features, storm surge	Long- or short-term storm history & storm surge levels	Risk of flooding (standing water)	
Is the environment appealing?	Composite	Environmental Aesthetics	Viewscape	Is the environment appealing to the property owner?	Color of the water, wave sounds & movement, ocean horizon, sandy beaches	No metric or indicator of site appeal developed yet.	No metric or indicator of site appeal developed yet.	Site Appeal

Table S2: FEGS and metrics for Ornamental Extractors.

**Beneficiary Category**	14.02 Commercial/ Industrial	**Sub-Category**	14.0202 Timber, Fiber and Ornamental Extractors
**General Beneficiary Description**	Ornamental Extractors for organisms used for show, living in aquariums or used for jewelry/decorative products.		

**What Matters Directly to this Beneficiary?**	**Attribute 1**	**Attribute 2**	**Sub-Attribute (Fine Scale)**	**Desired Information**	**Ideal Biophysical Data (Underlying Desired Information)**	**Available Data (and Unit)**	**Translation of Ideal Biophysical Data to Desired Information**	**Metric**
Is this a good place to look for ornamental exports?	Water	Water Movement	Currents	If in a boat, do I have to anchor?	Tide, weather, wind speed & direction	Tides, wind speed & direction	Marine advisory report based on wind speed, direction, tides	Flow Rate
			Wave intensity	Is it safe to go out?	Weather, wind speed & direction	Wind speed & direction	Marine advisory report based on wind speed, direction, wave height	Wave Height
	Fauna	Commercially important fauna	Aquarium Species	Will I find what I am expecting?	Species, size, abundance, diversity, health, specific look	Species, size, abundance, diversity	Presence of species is directly translated	Presence & Abundance
			Organisms used for products	Are the materials there and are they usable?	Species, size, abundance, health, specific look	Species, size, abundance, diversity	Presence of species is directly translated	Presence & Abundance
	Flora	Commercially important flora	Aquarium Species	Will I find what I am expecting?	Species, size, abundance, diversity, health, specific look	Species, size, abundance, diversity	Presence of species is directly translated	Presence & Abundance
			Organisms used for products	Are the materials there and are they usable?	Species, size, abundance, health, specific look	Species, size, abundance, diversity	Presence of species is directly translated	Presence & Abundance
	Soil & Substrate	Substrate quality	Reef Structure	Are the corals more desirable due to the reef complexity?	Morphology	Morphology, species	Complex reef structure increases appealing structure of corals for jewelry or décor	topographic complexity

Table S3: FEGS and metrics for Pharmaceutical Extractors.

**Beneficiary Category**	14.02 Commercial/ Industrial	**Sub-Category**	14.0207 Pharmaceutical and Food Supplement Suppliers (bioprospectors or the people who use the drugs, or other products?).
**General Beneficiary Description**	Organisms used for medical, cosmetics and beauty products		

**What Matters Directly to this Beneficiary?**	**Attribute Tier 1**	**Attribute Tier 2**	**Sub-Attribute**	**Desired Information**	**Ideal Biophysical Data (Underlying Desired Information)**	**Available Data (and Unit)**	**Translation of Ideal Biophysical Data to Desired Information**	**Metric**
Is this a good place to look for natural products?	Water	Water movement	Currents	If in a boat, do I have to anchor?	Tide, weather, wind speed & direction	Tides, wind speed & direction	Marine advisory report based on wind speed, direction, tides	Flow rate
			Wave intensity	Is it safe to go out?	Weather, wind speed & direction	Wind speed & direction	Marine advisory report based on wind speed, direction, wave height	Wave Height
	Fauna	Medicinal fauna	Presence	Are the materials there and are they usable?	Abundance, health, size, species	Abundance, health, size, species	Presence of species is directly translated	Presence & Abundance
		Commercially important fauna	Presence	Are the materials there and are they usable?	Abundance, health, size, species	Abundance, health, size, species	Presence of species is directly translated	Presence & Abundance
		Fauna Community	Diversity	Is there a chance of novel relationships for new bioprospecting?	Diversity, richness	Diversity, richness	Competition increases chance of finding new drug potential	Diversity
	Flora	Medicinal flora	Presence	Are the materials there and are they usable?	Abundance, health, size, species	Abundance, health, size, species	Presence of species is directly translated	Presence & Abundance
		Commercially important flora	Presence	Are the materials there and are they usable?	Abundance, health, size, species	Abundance, health, size, species	Presence of species is directly translated	Presence & Abundance
		Floral Community	Diversity	Is there a chance of novel relationships for new bioprospecting?	Diversity, richness	Diversity, richness	Competition increases chance of finding new drug potential	Diversity

Table S4: FEGS and metrics for Aquaculturists.

**Beneficiary Category**		14.01 Agriculture			**Sub-Category**		14.0105 Aquaculturists	
**General Beneficiary Description**	These beneficiaries farm aquatic fauna, most likely be used for rearing and transplanting juvenile corals.							
**What Matters Directly to this Beneficiary?**	**Attribute 1**	**Attribute 2**	**Sub-Attribute (Fine Scale)**	**Desired Information**	**Ideal Biophysical Data (Underlying Desired Information)**	**Available Data (and Unit)**	**Translation of Ideal Biophysical Data to Desired Information**	**Metric**
Is this a good area to grow coral nurseries?	Water	Water Quality	Water Clarity	Is there sufficient visibility to be healthy for coral growth?	Turbidity values, Secchi disk, Satellite imagery, light meter	Turbidity: FTU & NTU, ppm. Visibility: m. Satellite chlorophyll a: relative concentrations. Light penetration: Kd, PAR	Insufficient growth could reflect too much sedimentation	Light Penetration
			Chemicals & Contaminants	Is WQ sufficient to grow juvenile corals?	Contaminants	Coliforms, enterococci, vibrios (CFUs). Microbial toxins, heavy metals & chemicals: µmol l−1)	Insufficient growth could reflect bad water quality	Water Quality Standards
			Temperature	Is temp sufficient to grow juvenile corals?	Temperature	Temperature	Insufficient growth could reflect unideal temperatures	Temperature
		Water Movement	Currents	If in a boat, do I have to anchor?	Tide, weather, wind speed & direction	Tides, wind speed & direction	Marine advisory report based on wind speed, direction, tides	Flow rate
				Is there sufficient flushing for coral growth?	Tide, weather, wind speed & direction	Tides, wind speed & direction	Marine advisory report based on wind speed, direction, tides	Flow rate
			Wave Intensity	Is it safe to go out?	Weather, wind speed & direction	Wind speed & direction	Marine advisory report based on wind speed, direction, wave height	Wave Height
	Flora	Flora Community	Presence	Will algae overgrow juvenile corals?	Abundance	Abundance	Too much algae will overgrow corals and ideal open substrate	Abundance

(Table S4. Continued)

What Matters Directly to this Beneficiary?	Attribute 1	Attribute 2	Sub-Attribute (Fine Scale)	Desired Information	Ideal Biophysical Data (Underlying Desired Information)	Available Data (and Unit)	Translation of Ideal Biophysical Data to Desired Information	Metric
Is this a good area to grow coral nurseries?	Soil & Substrate	Substrate Quantity	Not defined	Is there enough substrate to transplant new corals onto or need room for man-made structures	% Coral Cover	% Coral Cover	More open substrate means more room to settle but also if using man made structure, you might want less substrate	% Cover
		Substrate Quality	Not defined		Reef type, % Cover	Reef type, % Cover	Corals grow best on certain types of substrate	% Cover

Table S5: FEGS and metrics for kayakers, paddleboarders and boaters.

**Beneficiary Category**	00.06 Recreational				**Sub-Category**	00.0606 Boaters		
**General Beneficiary Description**	Kayaks, Paddleboarders, and Boaters who will not be in the water.							
**What Matters Directly to this Beneficiary?**	**Attribute 1**	**Attribute 2**	**Sub-Attribute (Fine Scale)**	**Desired Information**	**Ideal Biophysical Data (Underlying Desired Information)**	**Available Data (and Unit)**	**Translation of Ideal Biophysical Data to Desired Information**	**Metric**
Is this a good place to go boating?	Water	Water Quality	Visibility	Is there sufficient visibility to see coral reef from glass bottom boats or in kayaks?	Turbidity values, Secchi disk, Satellite imagery, light meter	Turbidity: FTU & NTU, ppm. Visibility: m. Satellite chlorophyll a: relative concentrations. Light penetration: Kd, PAR	Glass bottom boat tours will depend on visibility	Visibility
		Water Movement	Currents	If in a boat, do I have to anchor?	Tide, weather, wind speed & direction	Tides, wind speed & direction	Marine advisory report based on wind speed, direction, tides	Flow rate
			Wave intensity	Is it safe to go out? Does boat captain need to be of a certain level of experience?	Wave height, speed & direction	Wave height, speed & direction	Marine advisory report based on wave speed, direction, height	Wave Height
	Fauna	Charismatic Fauna	Fish Taxa	Will I see what im expecting or any interesting animals?	Large, colorful and unusual marine organisms	Presence, abundance	Presence of fauna directly increases appeal	Species & Presence
	Composite	Environmental Aesthetics	Viewscape	Is this reef aesthetically enjoyable?	Color of water, algae, clarity & smell, lack of sound	Field crew opinion, secchi depth, algal abundance	Opinion of field crew reflects angler’s preference	Site Appeal

Table S6: FEGS and metrics for Non-Users.

Beneficiary Category		14.09 Non-Use		Sub-Category				
General Beneficiary Description		People who care about the overall health of the reef but are not ever planning on visiting a reef						
**What Matters Directly to this Beneficiary?**	**Attribute 1 (Coarse Scale)**	**Attribute 2 (Coarse Scale)**	**Sub-Attribute (Fine Scale)**	**Desired Information**	**Ideal Biophysical Data (Underlying Desired Information)**	**Available Data (and Unit)**	**Translation of Ideal Biophysical Data to Desired Information**	**Metric**
Is the coral reef the way it's supposed to be?	Water	Water Quality	Chemicals and contaminants	Is the WQ good enough for a healthy reef?	Fecal matter, pathogens, & toxins	Coliforms, enterococci, vibrios (CFUs). Microbial toxins, heavy metals & chemicals: $\mu mol\ l^{-1}$)	Use EPA recommended standards to translate into necessary water quality for reef function	Water Quality Standard pass/fail
	Fauna	Fauna Community	Diversity	Is the diversity high enough for a pretty and functional reef?	% live coral cover	% live coral cover	Community metrics translate to overall reef function	% live coral cover
			Abundance	Is the abundance high enough for a pretty and functional reef?	% live coral cover and abundance	% live coral cover and abundance	Community metrics translate to overall reef function	% live coral cover and abundance
			Nuisance Species	Are these species present and do they effect the reef health?	Abundance	Abundance	Community metrics translate to overall reef function	Abundance
		Charismatic Fauna	Presence/absence	Do these species matter to the beneficiary?	Large, colorful and unusual marine organisms	Presence, abundance	Presence of fauna directly increases appeal	Presence

(Table S6. Continued)

What Matters Directly to this Beneficiary?	Attribute 1 (Coarse Scale)	Attribute 2 (Coarse Scale)	Sub-Attribute (Fine Scale)	Desired Information	Ideal Biophysical Data (Underlying Desired Information)	Available Data (and Unit)	Translation of Ideal Biophysical Data to Desired Information	Metric
Is the coral reef the way it's supposed to be?	Flora	Flora Community	Diversity	Is the diversity high enough for a pretty and functional reef?	% cover	% cover	Community metrics translate to overall reef function	% cover
			Abundance	Is the abundance high enough for a pretty and functional reef?	% cover and abundance	% cover and abundance	Community metrics translate to overall reef function	% cover and abundance
			Nuisance Species	Are these species present and do they effect the reef health?	Presence/absence	Presence/absence	Community metrics translate to overall reef function	Presence
	Soil & Substrate	Substrate quality	Reef Structure	Does the amount of structure look nice and support reef function?	Reef complexity, grooves & spurs, swim throughs, caves	Reef type, rugosity	Unique & complex reef structure adds to wow factor & grandeur	Number & and size of underwater features & topographic complexity
	Composite	Naturalness	Ecological Condition	Is the reef functioning normally?	health and function	Health indices	Health score directly translates	Health Grade, BCG
Is the environment appealing?	Composite	Environmental Aesthetics	Viewscape	Does the reef look nice?	Colors, shapes, diversity, movement	No indicator or metric for site appeal has been developed	Unique & complex reef viewscape adds to wow factor & grandeur. large amounts of weedy species overgrowing corals are unappealing	Site Appeal

Table S7: FEGS and metrics for Learners.

Beneficiary Category	14.08 Learners	Sub-Category
**General Beneficiary Description**	People who care about the overall health of the reef and are also studying specific aspects of the reef including assessment, measurement and monitoring: educators, researchers	

What Matters Directly to this Beneficiary?	Attribute 1 (Coarse Scale)	Attribute 2 (Coarse Scale)	Sub-Attribute (Fine Scale)	Desired Information	Ideal Biophysical Data (Underlying Desired Information)	Available Data (and Unit)	Translation of Ideal Biophysical Data to Desired Information	Metrics
Can we learn from this coral reef?	Water	Water Movement	Currents	If in a boat, do I have to anchor?	Tide, weather, wind speed & direction	Tides, wind speed & direction	Marine advisory report based on wind speed, direction, tides	Flow Rate
			Wave intensity	Is it safe to go out? Does boat captain need to be of a certain level of experience?	Wave height, speed & direction	Wave height, speed & direction	Marine advisory report based on wave speed, direction, height	Wave Height
		Water Quality	Visibility	Is the clarity good enough for a healthy reef?	Turbidity values, Secchi disk, Satellite imagery, light meter	Turbidity: FTU & NTU, ppm. Visibility: m. Satellite chlorophyll a: relative concentrations. Light penetration: Kd, PAR	Secchi disk measurements directly translate to visibility, but turbidity & light penetration need to be translated to distances in ft or m	Light Penetration
			Chemicals and contaminants	Is the WQ good enough for a healthy reef? Is WQ sufficient to be safe for contact?	Fecal matter, pathogens, & toxins	Coliforms, enter-ococci, vibrios (CFUs). Microbial toxins, heavy metals & chem-icals (μmol l^{-1})	Use EPA recommended standards to translate into necessary water quality for reef function	Water Quality Standard pass/fail

(Table S7. Continued)

What Matters Directly to this Beneficiary?	Attribute 1 (Coarse Scale)	Attribute 2 (Coarse Scale)	Sub-Attribute (Fine Scale)	Desired Information	Ideal Biophysical Data (Underlying Desired Information)	Available Data (and Unit)	Translation of Ideal Biophysical Data to Desired Information	Metric
Can we learn from this coral reef?	Fauna	Fauna Community	Diversity	Is the diversity high enough for a pretty and functional reef?	% live coral cover, richness	% live coral cover, richness	Community metrics translate to reef function. Specific species are present for researchers to study them.	% Live Coral Cover
	Fauna	Fauna Community Charismatic Fauna	Abundance	Is the abundance high enough for a pretty and functional reef?	% live coral cover and abundance	% live coral cover and abundance	Community metrics translate to reef function. Specific species are present for researchers to study them.	Abundance
			Nuisance Species	Are these species present and do they effect the reef health?	Abundance	Abundance		Presence & Abundance
			Abundance	Do these species attract the beneficiary?	Large, colorful and unusual marine organisms	Presence, abundance		Presence & Abundance
		Keystone Fauna	Abundance	Are these species present and do they represent the reef health?	Abundance	Abundance	Presence of keystone fauna indicates a healthy reef	Presence
		Rare Fauna	Abundance	Are these species present and do they represent the reef health?	Abundance	Abundance	Presence of rare fauna indicates a healthy reef	Presence
	Flora	Flora Community	Diversity	Is the diversity high enough for a pretty and functional reef?	% cover, richness	% cover, richness	Community metrics translate to reef function. Specific species are present for researchers to study them.	% Cover
			Abundance	Is the abundance high enough for a pretty and functional reef?	% cover, abundance	% cover, abundance	Community metrics translate to reef function. Specific species are present for researchers to study them.	Abundance

(Table S7. Continued)

What Matters Directly to this Beneficiary?	Attribute 1 (Coarse Scale)	Attribute 2 (Coarse Scale)	Sub-Attribute (Fine Scale)	Desired Information	Ideal Biophysical Data (Underlying Desired Information)	Available Data (and Unit)	Translation of Ideal Biophysical Data to Desired Information	Metric
Can we learn from this coral reef?	Flora	Flora Community	Nuisance Species	Are these species present and do they effect the reef health?	Abundance	Abundance	Community metrics translate to overall reef function. Researchers that care about certain species need to be able to find them to study them.	Presence & Abundance
		Keystone Fauna	Abundance	Are these species present and do they represent the reef health?	Abundance	Abundance	Presence of keystone flora indicates a healthy reef	Presence
		Rare Fauna	Abundance	Are these species present and do they represent the reef health?	Abundance	Abundance	Presence of rare flora indicates a healthy reef	Presence
	Composite	Naturalness	Ecological Condition	Is the reef functioning normally?	health and function	Health indices	Health score directly translates	Health Grade, BCG
	Soil & Substrate	Substrate quality	Reef Structure	Does the amount of structure look nice and support reef function?	Reef complexity, grooves & spurs, swim throughs, caves	Reef type, rugosity	Unique & complex reef structure adds to wow factor & grandeur	Number & size of underwater features & topographic complexity

Table S8: FEGS and metrics for Inspirational Beneficiary.

**Beneficiary Category**	14.07 Inspirational	**Sub-Category**	14.0701 Spiritual and Ceremonial, 14.0702 Artists
**General Beneficiary Description**	People who care about the overall health of the reef; artists (from photos/videos), cultural and spiritual and ceremonial.		

**What Matters Directly to this Beneficiary?**	**Attribute 1 (Coarse Scale)**	**Attribute 2 (Coarse Scale)**	**Sub-Attribute (Fine Scale)**	**Desired Information**	**Ideal Biophysical Data (Underlying Desired Information)**	**Available Data (and Unit)**	**Translation of Ideal Biophysical Data to Desired Information**	**Metrics**
Is this reef inspirational?	Water	Water Movement	Currents	If in a boat, do I have to anchor?	Tide, weather, wind speed & direction	Tides, wind speed & direction	Marine advisory report based on wind speed, direction, tides	Flow Rate
			Wave intensity	Is it safe to go out? Does boat captain need to be of a certain level of experience?	Wave height, speed & direction	Wave height, speed & direction	Marine advisory report based on wave speed, direction, height	Wave Height
		Water Quality	Visibility	Is the clarity good enough for a healthy reef?	Turbidity values, Secchi disk, Satellite imagery, light meter	Turbidity: FTU & NTU, ppm. Visibility: m. Satellite chlorophyll a: relative concentrations. Light penetration: Kd, PAR	Secchi disk measurements directly translate to visibility, but turbidity & light penetration need to be translated to distances in ft or m	Light Penetration
			Chemicals and contaminants	Is the WQ good enough for a healthy reef? Is WQ sufficient to be safe for contact?	Fecal matter, pathogens, & toxins	Coliforms, enterococci, vibrios (CFUs). Microbial toxins, heavy metals & chemicals: µmol l^{-1})	Use EPA recommended standards to translate into human health risks	Water Quality Standard pass/fail

(Table S8. Continued)

What Matters Directly to this Beneficiary?	Attribute 1 (Coarse Scale)	Attribute 2 (Coarse Scale)	Sub-Attribute (Fine Scale)	Desired Information	Ideal Biophysical Data (Underlying Desired Information)	Available Data (and Unit)	Translation of Ideal Biophysical Data to Desired Information	Metric
Is this reef inspirational?	Fauna	Fauna Community	Diversity	Is the diversity high enough for a pretty and functional reef?	% live coral cover, richness	% live coral cover, richness	Community metrics translate to overall reef function	% Live Coral Cover
			Abundance	Is the abundance high enough for a pretty and functional reef?	% live coral cover, abundance	% live coral cover, abundance	Community metrics translate to overall reef function	Abundance
			Nuisance Species	Are these species present and do they effect the reef health?	Abundance	Abundance	Community metrics translate to overall reef function	Presence & Abundance
		Charismatic fauna	Abundance	Do these species attract the beneficiary?	Large, colorful and unusual marine organisms	Presence, abundance	Presence of fauna directly increases appeal	Presence & Abundance
		Spiritually/ culturally important fauna	Abundance	Do these species attract the beneficiary?	Abundance	Abundance	These species are meaningful to the community and their presence increases appeal	Presence & Abundance
	Flora	Flora Community	Diversity	Is the diversity high enough for a pretty and functional reef?	% cover, richness	% cover, richness	Community metrics translate to overall reef function	% Cover
			Abundance	Is the abundance high enough for a pretty and functional reef?	% cover, abundance	% cover, abundance	Community metrics translate to overall reef function	Abundance

(Table S8. Continued)

What Matters Directly to this Beneficiary?	Attribute 1 (Coarse Scale)	Attribute 2 (Coarse Scale)	Sub-Attribute (Fine Scale)	Desired Information	Ideal Biophysical Data (Underlying Desired Information)	Available Data (and Unit)	Translation of Ideal Biophysical Data to Desired Information	Metric
Is this reef inspirational?	Flora	Flora Community	Nuisance Species	Are these species present and do they effect the reef health?	Abundance	Abundance	Community metrics translate to overall reef function	Presence & Abundance
		Spiritually/ culturally important flora	Abundance	Do these species attract the beneficiary?	Abundance	Abundance	These species are meaningful to the community and their presence increases appeal	Presence & Abundance
	Composite	Naturalness	Ecological Condition	Is the reef functioning normally?	health and function	Health indices	Health score directly translates	Health Grade, BCG
Is the environment appealing?	Composite	Environmental Aesthetics	Viewscape	Is there interesting enough viewscape to entertain divers?	Colors, shapes, diversity, movement	No indicator or metric for site appeal has been developed yet.	Unique & complex reef viewscape adds to wow factor & grandeur. large amounts of weedy species overgrowing corals are unappealing	Site Appeal
